# Quercetin Inhibits Biofilm Formation by Decreasing the Production of EPS and Altering the Composition of EPS in *Staphylococcus epidermidis*

**DOI:** 10.3389/fmicb.2021.631058

**Published:** 2021-03-04

**Authors:** Yongqi Mu, Hong Zeng, Wei Chen

**Affiliations:** ^1^Key Laboratory of Protection and Utilization of Biological Resources in Tarim Basin of Xinjiang Production and Construction Corps, Tarim University, Alar, China; ^2^College of Life Sciences, Tarim University, Alar, China; ^3^College of Animal Sciences Tarim University, Alar, China; ^4^Key Laboratory of Tarim Animal Husbandry and Science Technology of Xinjiang Production and Construction Corps, Tarim University, Alar, China; ^5^Engineering Laboratory for Tarim Animal Diseases Diagnosis and Control of Xinjiang Production and Construction Corps, Tarim University, Alar, China

**Keywords:** quercetin, biofilm, hydrophobicity, *ica*, PIA, *Staphylococcus epidermidis*

## Abstract

*Staphylococcus epidermidis* is an opportunistic pathogen, and its biofilm formation ability is an important virulent factor. Quercetin, a typical flavonoid ubiquitously used in dietary supplementation, is known for its antioxidant property, but its anti-biofilm activity against *S. epidermidis* remains unknown. In this study, the anti-biofilm activity of quercetin was investigated using *S. epidermidis* ATCC35984, a strong biofilm-positive strain. An attempt was made to disclose the mechanisms of the anti-biofilm activity of quercetin. *S. epidermidis* exhibited a less cell surface hydrophobicity after quercetin treatment. Also, quercetin effectively inhibited *S. epidermidis* cells from adhering to the glass slides. Quercetin downregulated the intercellular adhesion (*ica*) locus and then polysaccharide intercellular adhesin (PIA) production was reduced. Therefore, *S. epidermidis* cells became less hydrophobic, which supported quercetin’s anti-biofilm effect. Our study suggests that quercetin from plants be given further attention as a potential anti-biofilm agent against the biofilm formation of *S. epidermidis*, even biofilm infections of other bacteria.

## Introduction

*Staphylococcus epidermidis* is the most frequently encountered coagulase-negative *Staphylococci* (CoNS) species on human skin. Characteristically, the diseases caused by *S. epidermidis* and other CoNS are chronic and re-occur, which contrasts the potential of *S. aureus* to cause acute infections (Lowy and Franklin, 1998). Epidemiological studies have demonstrated the presence of the genus *Staphylococcus*, *Staphylococcus aureus*, and *S. epidermidis*, in approximately 50% of cases of bovine mastitis (Mello et al., 2020). The consequences of mastitis include economic losses due to the costs of treatment, lower milk production, changes in product quality, and culling (Halasal et al., 2007). Besides the economic losses, mastitis is a public health hazard since it can cause zoonoses and food poisoning (Fernandes et al., 2011; Gomes et al., 2016). *S. epidermidis* is notorious in particular for causing infections on indwelling medical devices, including cardiac implantable electric device (CIED) infection (Okada et al., 2021) and orthopedic device-related infection (Thompson et al., 2020), in which the pathogenesis usually involves biofilm formation. Moreover, the antibiotic therapy against pathogenic bacteria is currently decreasing, which is partly attributed to biofilm formation (Al-Yousef et al., 2017; Singh et al., 2017). Consequently, the ability of biofilm formation by *S. epidermidis* is focused on in recent years (Vadyvaloo and Otto, 2005; Xie et al., 2019; Mu et al., 2020).

It was observed that extracts of various plants and secondary metabolites isolated from plants such as quercetin, caffeine, menthol, and chlorogenic acid have demonstrated varying levels of biofilm inhibition in gram-negative pathogens (Choo et al., 2006; Annapoorani et al., 2012; Al-Yousef et al., 2017; Satish et al., 2017; Du et al., 2018). Quercetin is a natural flavonoid antioxidant (El-Khawagah et al., 2020; Rojas et al., 2020) able to scavenge reactive species and hydroxyl radicals (Boots et al., 2008), and it bears pharmaceutical significance including anticarcinogenic (Pereira et al., 1996), anti-inflammatory (Guardia et al., 2001), and antimicrobial properties (Nitiema et al., 2012). Although the anti-biofilm/anti-virulence effects of quercetin have been explored to a wider extent in both gram-positive and gram-negative as well as a few fungi species, its anti-biofilm efficacy against *Staphylococcus epidermidis* has not yet been reported.

In this study, we performed scanning electron microscopy (SEM), matrix components analysis, and a hydrophobic assay to investigate the effect and mechanisms of quercetin on *S. epidermidis* biofilm formation.

## Materials and Methods

### Bacterial Strains and Growth Conditions

*S. epidermidis* ATCC 35984 [intercellular adhesion (*ica*)-positive], a strong biofilm-positive strain, was used in this study. Unless specified otherwise, tryptic soy agar/broth (TSA/TSB; Becton Dickinson, 211825) were used to culture cells at 37°C overnight. OD_590_ was measured using a spectrophotometer (Bio-Rad) for cell growth. Each experiment was performed using at least three independent cultures.

### Assay for Biofilm Inhibition

A static biofilm formation assay was performed using 96-well polystyrene plates as previously described, with slight modifications (Pratt and Kolter, 1998; Xie et al., 2019; Mu et al., 2020). Briefly, cells were diluted 1:100 with fresh TSB and cultured with different concentrations of quercetin (0–1,000 μg ml^–1^) for 24 h without shaking at 37°C. Biofilms were stained with crystal violet (Sigma, C3886) and dissolved in 95% ethanol (0.5%, *w*/*v*). The optical density was measured at 490 nm in an enzyme-linked immunosorbent assay reader (Bio-Rad). Cell growth in the 96-well plates was also detected at OD_590_. Relative ability of biofilm formation was indicated as percent Relative Biofilm Formation (%) (RBF%), calculated by the following formula: RBF% = Treated OD_490_/Untreated OD_490_ × 100%. Each data point was averaged from at least 12 replicate wells (four wells from each of at least three independent cultures).

### Microscopic Visualization

Biofilms grown on glass slides were stained with crystal violet and were visualized by light microscopy (Nikon Eclipse Ti 100) at a magnification of ×400 (Nithyanand et al., 2010; Xie et al., 2019; Mu et al., 2020). SEM was used to observe biofilm cells as previously described (Lee et al., 2016). Briefly, *S. epidermidis* strain ATCC35984 cells were diluted 1:100 with fresh TSB and inoculated onto a coverslip (22 × 22 mm^2^) in the presence of quercetin (125 μg ml^–1^) at 37°C for 24 h without shaking.

### Effect of Quercetin on the Production and Components of Exopolysaccharides in *S. epidermidis*

To quantify the exopolysaccharides (EPS) produced by *S. epidermidis*, the cells were diluted 1:100 with fresh TSB and cultured for 24 h with shaking at 37°C. Then, the water extraction and alcohol precipitation method was used to collect EPS as previously described, with slight modifications (Petit and Pinilla, 1995; Li et al., 2011; Jin and Zhao, 2014), and air-dried at room temperature. The crude polysaccharides were dissolved by 0.5 mol L^–1^ hydrochloric acid and 121°C heat treated for 15 min. The EPS production of *S. epidermidis* was quantified using the degrees Brix assay (Ball, 2006) with or without treatment with quercetin (125 μg ml^–1^).

To detect the effect of quercetin on the components of EPS, the water extraction and alcohol precipitation method was used to collect EPS in the *S. epidermidis* culture, as above. Proteinase K and *n*-butyl alcohol (5:1, BOC Sciences, 71-36-3) were used to remove the proteins as described previously (Li et al., 2011). Following dialysis with distilled water overnight, the aqueous layer was collected. The liquid was lyophilized as an EPS sample for use.

Pre-column derivation high-performance liquid chromatography (HPLC) was used to detect the monosaccharide composition of EPS in *S. epidermidis* (Zhang et al., 2009; Xie et al., 2019; Mu et al., 2020). Ribose (∼1 mmol, per 50 ml) was used as the internal standard solution. A mixture of mannose, glucosamine, rhamnose, glucuronic acid, galacturonic acid, galactosamine, arabinose, glucose, galactose, xylose, and fucose (∼0.1 mmol of each monosaccharide; Sigma) was dissolved in water, followed by adding 5.0 ml of the internal standard solution. The mixture solution was then diluted to 50 ml and retained for 1-pheny-3-methyl-5-pyrazolone (PMP; Macklin, P816062) derivation.

The chromatographic conditions were generally as follows: column, Eclipse XDB-C^18^; temperature, 25°C; solvent, 0.4% triethylamine in 20 mmol L^–1^ ammonium acetate buffer solution (pH 6.3 with acetic acid)–acetonitrile (83:17); and flow rate, 1 ml min^–1^. The eluate was monitored at 245 nm.

The correction factor for each monosaccharide (*f*_i/s_) and the content of every monosaccharide in the polysaccharide hydrolysis solution (*W*) was calculated using the equations *f*_i/s_ = (*W*_i_/*W*_s_)/(*A*_i_/*A*_s_) and *W* = *f*_i/s_(*A*_i_/*A*_s_)*W*_s_, respectively. *A*_s_ and *A*_i_ are the peak areas of the internal ribose standard and the standard monosaccharide in the reference solution, respectively. *W*_s_ and *W*_i_ are the contents of the internal ribose standard and the standard monosaccharide in the reference solution, respectively.

### Cell Surface Hydrophobicity Assay

Cell surface hydrophobicity was tested as previously described (Rosenberg et al., 1980; Xie et al., 2019; Mu et al., 2020). Briefly, 1 ml of bacteria (OD_400_ = 0.6) was placed into glass tubes and 250 μl of *n*-hexadecane (Macklin, H810865) was added. The decrease in the OD_400_ of the aqueous phase was taken as a measure of *H*%, which was calculated with the formula: *H*% = [(OD_0_ - OD)/OD_0_] × 100, where OD_0_ and OD are the OD_400_ before and after extraction with *n*-hexadecane, respectively. The experiments were performed using three independent cultures per condition.

### Quantitative Real-Time RT-PCR Assay

To explore further the possible mechanisms of the inhibition against *S. epidermidis* biofilm by quercetin, quantitative reverse transcription PCR (qRT-PCR) was performed to investigate the transcription levels of several biofilm-associated genes in *S. epidermidis* ATCC35984 with and without quercetin treatment. Gene-specific primers were used for these genes and *gyrB* used as a housekeeping control (Table 1). The expression level of the housekeeping gene *gyrB* was used to normalize the expression data of the genes of interest. The qRT-PCR method was adapted from a previous study (Wang et al., 2011). qRT-PCR was performed using a SYBR green PCR master mix (TransGen Biotech) and an ABI PRISM 7500 Real-Time PCR System (Rotor-Gene Q) with two independent cultures. All experiments were performed in triplicate. The 2^–△△Ct^ method was used to analyze the quantitative real-time PCR data.

**TABLE 1:** Primers for quantitative reverse transcriptase PCR.

Genes	Primer sequences
*gyrB*	5′-TGACGAGGCATTAGCAGGTT-3′
	5′-GTGAAGACCGCCAGATACTTT-3′
*icaR*	5′-CATTGACGGACTTTACCAGTTTT-3′
	5′-ATCCAAAGCGATGTGCGTAG-3′
*icaB*	5′-GAAACAGGCTTATGGGACTTTG-3′
	5′-CAAGTGCGCGTTCATTTTT-3′

### Statistical Analysis

GraphPad Prism 5 was used to calculate the mean and the standard deviation of the mean. All experiments were performed in triplicate and the data obtained from the experiments were presented as mean values; the difference between the control and the tested groups were analyzed using Student’s *t*-test. Significant differences were *P* < 0.05.

## Results

### Quercetin Inhibited Biofilm Formation by *S. epidermidis* in a Dose-Dependent Manner

**FIGURE 1:**
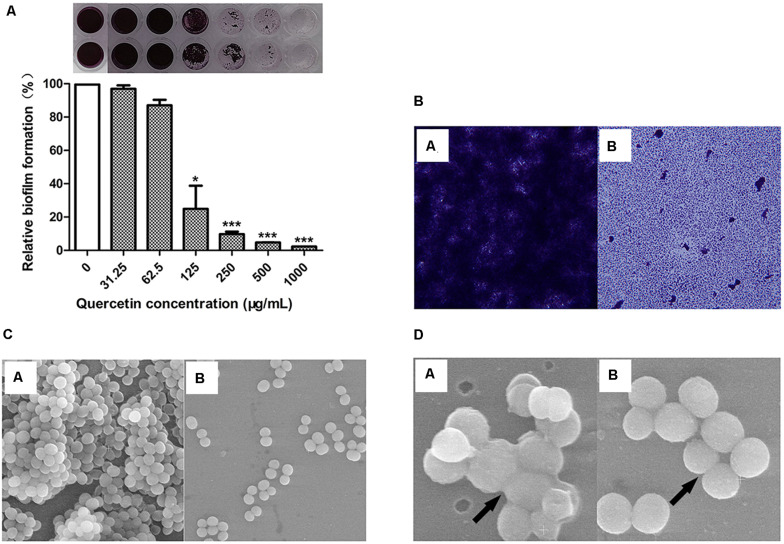
Quercetin significantly reduced *Staphylococcus epidermidis* (ATCC 35984) biofilm formation. The biofilm formation (OD_490_) of *S. epidermidis* **(A)** was quantified at different concentrations of quercetin (0–1,000 μg ml^–1^) at 37°C after 24 h in 96-well plates. The relative activity of biofilm formation was indicated as percent Relative Biofilm Formation (%) (RBF%), calculated by the following formula: RBF% = Treated OD_490_/Untreated OD_490_ × 100%. *Columns* represent the means for three independent experiments. *Error bars* indicate the standard deviations. Statistically significant differences (determined by Student’s *t*-test) are indicated as ***P < 0.001 and *P < 0.05 vs. the control group. Biofilms formed by *S. epidermidis* ATCC 35984 were visualized by light microscopy **(B)** and SEM **(C,D)**. SEM was used to examine the biofilm cells grown on coverslips in the presence of quercetin (125 μg ml^–1^). At least three independent experiments were conducted. *a*, untreated control; *b*, quercetin treated.

The results showed that quercetin inhibited the biofilm formation of *S. epidermidis* ATCC 35984 in a dose-dependent manner (Figure 1A). Specifically, it decreased the biofilm formation of *S. epidermidis* ATCC 35984 by ≥ 90% at 250 μg ml^–1^ and by ≥ 95% at 500 μg ml^–1^.

The change in biofilm formation with or without quercetin treatment was observed using light microscopy and SEM. The results showed that the biofilms treated with 125 μg ml^–1^ of quercetin became thinner, looser, and even easier to eradicate than the untreated biofilms (Figure 1B). SEM analysis revealed that fewer cells attached to the coverslips when treated with quercetin (Figure 1C). Additionally, fewer intercellular substances were present in the treated group compared with those in the untreated group (Figure 1D). No morphologic abnormality was observed in the presence of quercetin. The growth curves of *S. epidermidis* cells were also measured in the presence of quercetin (125 μg ml^–1^), and a decrease in cell growth was not observed (data not shown). The cell growth and microscopic results indicate that the inhibition by quercetin against *S. epidermidis* biofilm formation is attributed to anti-biofilm activity rather than antibacterial activity.

### Quercetin Decreased Cell Surface Hydrophobicity

**FIGURE 2:**
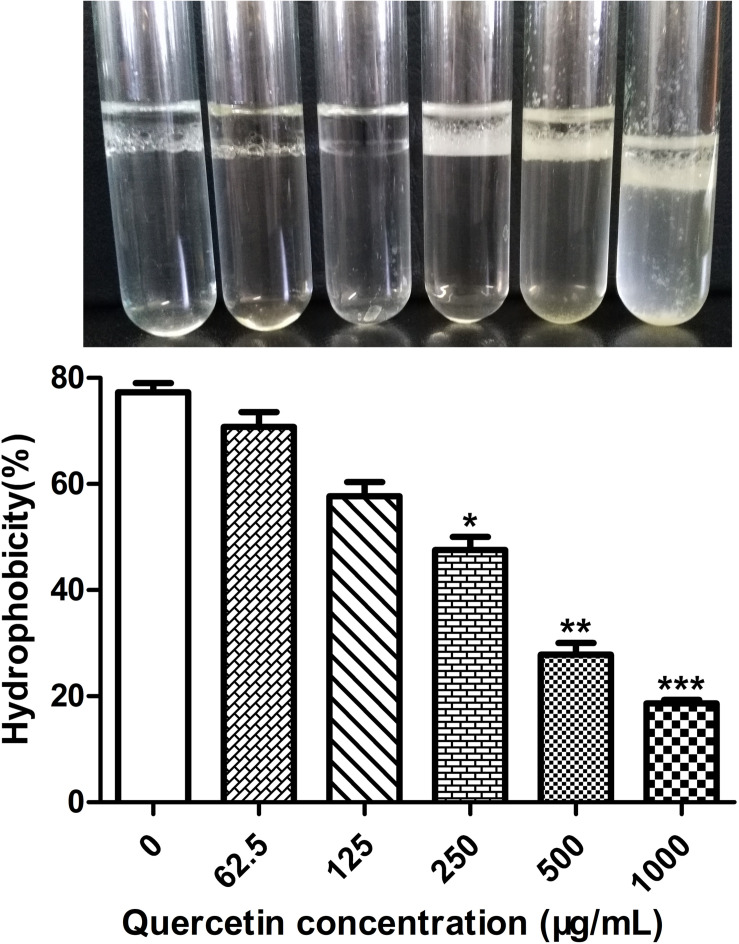
Effects of quercetin on the hydrophobicities of cell surfaces. *Staphylococcus epidermidis* ATCC 35984 cells were grown overnight and a hydrophobicity assay was performed by hexadecane extraction. Aqueous phase absorbance was measured at OD_400_, and high turbidity indicated less hydrophobicity. *Columns* represent the means for three independent experiments. *Error bars* indicate the standard deviations. Statistically significant differences (determined by Student’s *t*-test) are indicated as ***P < 0.001, **P < 0.01, and *P < 0.05 vs. the control group.

Surface hydrophobicity facilitates adherence to hydrophobic surfaces. Thus, it plays a crucial role in biofilm formation by *Staphylococci* (Lee et al., 2016; Xie et al., 2019). The cell surface hydrophobicity (CSH) assay was performed to explore the mechanism underlying the inhibition by quercetin against biofilm formation by *S. epidermidis*. The results showed that *S. epidermidis* cells became less hydrophobic when treated with quercetin (Figure 2), which at least partly demonstrates the inhibitory mechanisms of quercetin on reduced biofilm formation.

### Quercetin Decreased the Production of EPS and Altered the Composition of EPS Produced by *S. epidermidis*

**FIGURE 3:**
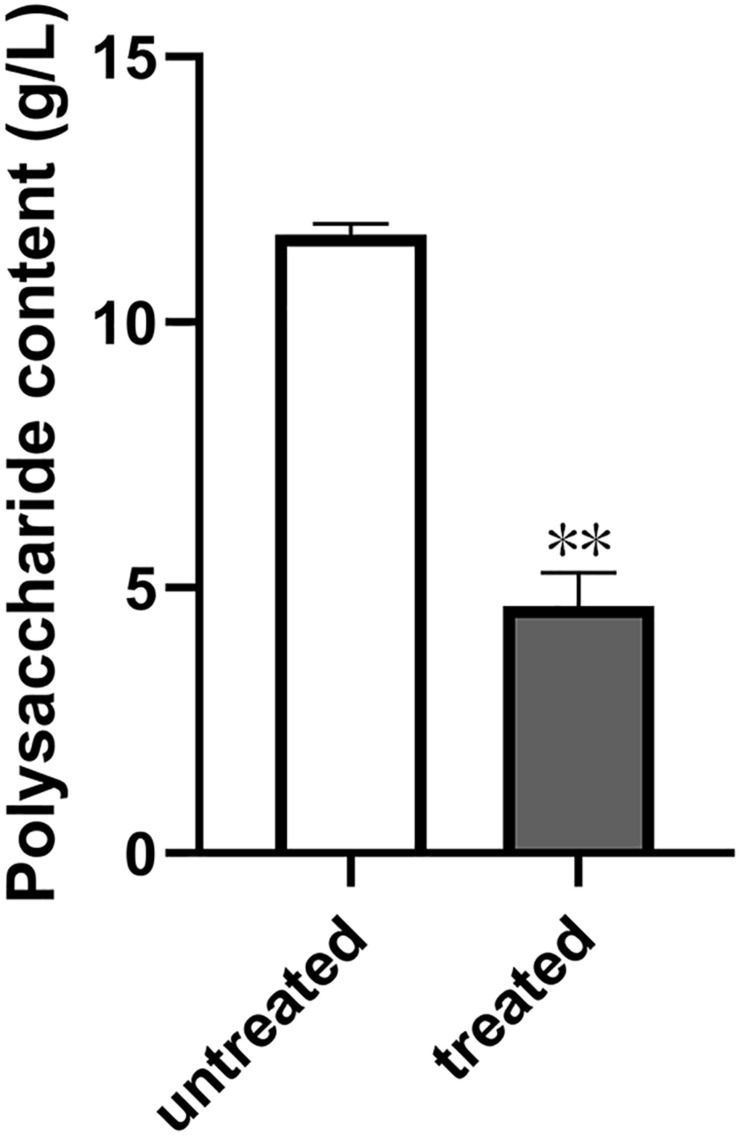
Effect of quercetin on the exopolysaccharide (EPS) production by *Staphylococcus epidermidis*. Degrees Brix was used to detect the content of EPS in *S. epidermidis* with or without quercetin treatment (125 μg ml^–1^). *Error bars* indicate the standard deviations. Statistically significant differences (determined by Student’s *t*-test) are indicated as **P < 0.01 vs. the control group.

**FIGURE 4:**
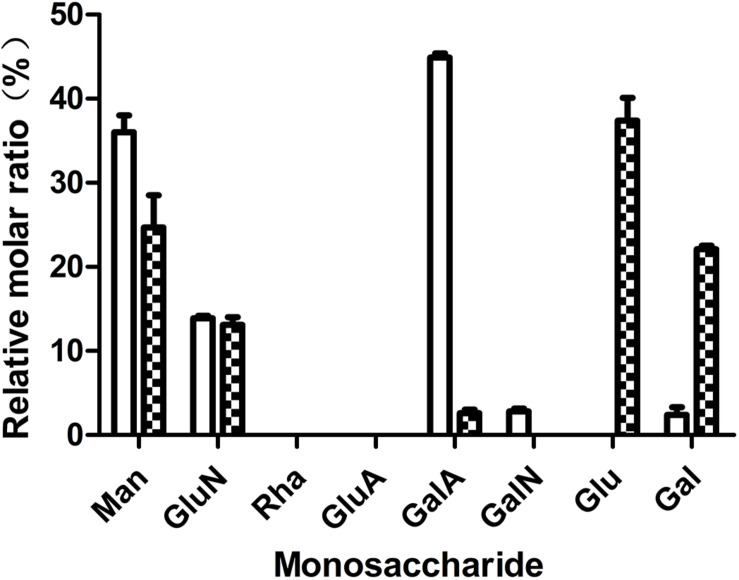
Effects of quercetin on the exopolysaccharide (EPS) components of *Staphylococcus epidermidis* ATCC 35984. The monosaccharides include mannose (Man), glucosamine (GluN), rhamnose (Rha), glucuronic acid (GluA), galacturonic acid (GalA), galactosamine (GalN), glucose (Glu), galactose (Gal), and arabinose (Ara). *Columns* represent the means for three independent experiments. *Error bars* indicate the standard deviations. *Open rectangle*, untreated; *filled rectangle*, treated.

In our previous work, we tested the dependent type of the biofilm formation of *S. epidermidis* ATCC 35984. It was found that biofilm formation by *S. epidermidis* ATCC 35984 mainly depends on EPS consisting of reductive polysaccharides in which the dihydroxy groups are unsubstituted (Xie et al., 2019; Mu et al., 2020). Thus, we detected the effect of quercetin on EPS. The results showed that the production of EPS by *S. epidermidis* ATCC 35984 was reduced when treated with quercetin (Figure 3). Specifically, for strain ATCC 35984 when treated with quercetin, galactosamine (GalN) was absent and glucose (Glu) obviously appeared in the monosaccharide composition compared with the control. Additionally, the proportion of galactose (Gal) was increased while the proportions of mannose (Man) and galacturonic acid (GalA) were decreased; especially GalA was significantly reduced (Figure 4).

### Quercetin Reduced PIA Production by Downregulated *ica* Locus

**FIGURE 5:**
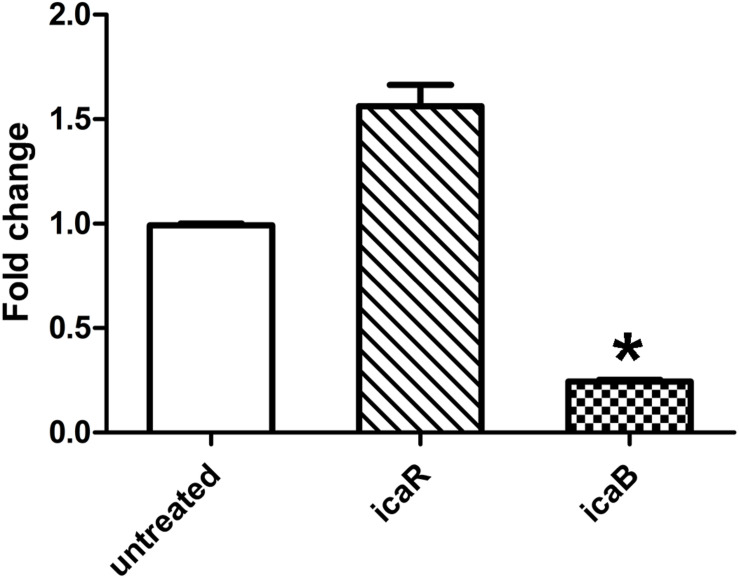
Relative mRNA expression (mean ± SEM) of *icaB* and *icaR* in *Staphylococcus epidermidis* with or without quercetin treatment (125 μg ml^–1^). Downregulation or upregulation of gene expression was considered significant when the relative expression was decreased or increased ≥ 2-fold. All fold changes have been normalized to *gyrB* as the reference gene. Data are the means of fold changes with standard deviations from three independent experiments amplified in triplicate. Error bars indicate the standard deviations. Statistically significant differences (determined by Student’s t-test) are indicated as *P < 0.05 vs. the control group.

As quercetin from plant extracts exhibited potent inhibition on the biofilm formation of *S. epidermidis* ATCC 35984, we were curious why it could decrease polysaccharide intercellular adhesin (PIA) production. Thus, the expression of the *ica* locus was analyzed. Quercetin treatment resulted in a downregulation of the *ica* locus which was associated with the cell adhesion in *S. epidermidis* biofilm formation (Figure 5). The decrease of cell-to-cell adhesion caused the reduction of biofilm formation.

## Discussion

*S. epidermidis* is the main reason for biofilm-associated infections. Unlike *S. aureus*, biofilm formation by *S. epidermidis* is an important and the sole virulence factor in the onset and development of device-related infections in clinical settings. Meanwhile, the emergence of multidrug resistance among clinical pathogens has been proven to be related to biofilm formation. Thus, novel antimicrobial and antifouling agents that cannot be surpassed by those pathogens are required. Increasing evidence of plant-derived molecules as novel antimicrobials against multidrug-resistant clinical pathogens show that local special plants may become promising candidates in which novel antibiotics could be found (Packiavathy et al., 2014; Kannappan et al., 2020).

In our previous study, we found that the extract of *Coreopsis tinctoria* Nutt, a special local plant in Xinjiang, obviously exhibited an inhibitory effect on biofilm formation in *S. epidermidis*. Also, we tested the components in *C. tinctoria* Nutt and found that quercetin is rich in this local plant. Thus, we assumed that quercetin has potential anti-biofilm activity; we then chose quercetin instead of the phytochemical as our experimental subject. In the current study, quercetin was evaluated for its potential to hamper the biofilm formation in *S. epidermidis*. In a plate incubation assay, quercetin exhibited a concentration-dependent reduction in biofilm formation. Quercetin reduced *S. epidermidis* biofilm formation up to 90.5% at the concentration of 250 μg ml^–1^ and up to 95.3% at the concentration of 500 μg ml^–1^. The above results are different from those of Gopu et al. (2015), who reported 13–72, 8–80, and 10–61% reductions in the biofilm formation of 3 g-negative food-borne bacteria, *Klebsiella pneumoniae*, *Pseudomonas aeruginosa*, and *Yersinia enterocolitica*, at different concentrations of 5–40 μg mL^–1^, respectively.

The mechanism of *S. epidermidis* biofilm formation is a complex process in which many factors are involved. Particularly, many macromolecules, such as extracellular proteins, environmental DNA (eDNA), and EPS, are the main components in biofilms (Xie et al., 2019). Among the above three substances, the production of EPS was mainly considered as a key factor which facilitates the initial attachment of *S. epidermidis*. Thus, the inhibition of EPS production may cause less biofilm formation. Our results showed that quercetin had no degradation effect on proteins and DNA (see Supplementary Figures S2A,B) in this study. However, a reduced production of EPS was observed in *S. epidermidis* when treated with quercetin (Figure 3). The above result is comparable with those of Abraham et al. (2011) and Gopu et al. (2015). Also, the composition of EPS produced by *S. epidermidis* changed with treatment of quercetin (Figure 4). Our previous work also showed that the composition of *S. epidermidis* EPS was altered after being treated with spent media from *Actinomycetes* (Xie et al., 2019; Mu et al., 2020). However, the changed composition of EPS is different. GalN was absent in quercetin treatment, while arabinose (Ara) and Gal were absent in the treatment of spent media from *Actinomycetes* compared with the control. GalN is a reductive glycoside that plays a crucial role in *S. epidermidis* biofilm formation (Mack et al., 1992; Rohde et al., 2005; Xie et al., 2019). Thus, GalN disappeared when treated with quercetin, which may cause a weaker biofilm formation. Additionally, the proportions of monosaccharides of EPS were significantly different. When treated with quercetin, Gal was increased while Man and GalA were decreased. These results suggest that quercetin acts on EPS in a different manner. Further investigation will be required to understand the mechanisms.

The biofilm formation of bacteria is significantly influenced by cell surface hydrophobicity. It has been reported that hydrophobic surfaces are preferred (Xie et al., 2019; Mu et al., 2020). Enzyme-like biofilm inhibitors and low-concentration antibiotics reduce the hydrophobicity of *S. epidermidis* cells (Xie et al., 2019; Mu et al., 2020). In this study, it appears that a decreased cell surface hydrophobicity attenuates the attachment of *S. epidermidis* cells to the plastic wells (Figure 1A), glass slides (Figure 1B), and coverslips (Figures 1C,D). The above findings suggest that the combination of antimicrobials with cell surface hydrophobicity reducers in clinical cases increases drug sensitivity and improves the therapeutic effect.

EPS produced by *S. epidermidis* is responsible for intercellular adhesion. PIA is a type of EPS. PIA production is catalyzed by four glucuronyltransferases encoded by the *icaADBC* operon (Liduma et al., 2012; Mu et al., 2020), which is negatively regulated by *icaR*, encoding a transcriptional repressor of the *icaADBC* operon (Conlon et al., 2002; Mu et al., 2020). The results of the relative gene expression, the downregulation of *icaB*, and the upregulation of *icaR* when treated with quercetin in the current study are in agreement with this conclusion. To further explore the effect of quercetin on biofilm, we used SEM and observed that intercellular substances were reduced (Figure 1D). Meanwhile, we tested the production of *S. epidermidis* exopolysaccharides using the degrees Brix assay. In agreement with the results of SEM, a significantly decreased production of exopolysaccharides in the treated groups was found (Figure 3).

## Conclusion

In conclusion, the results in this study indicate that quercetin exhibits anti-biofilm activity *via* decreasing PIA production and cell surface hydrophobicity. Quercetin warrants further attention as a potential biofilm inhibitor in biofilm-associated infections.

## Data Availability Statement

The original contributions presented in the study are included in the article/Supplementary Material, further inquiries can be directed to the corresponding author/s.

## Author Contributions

WC, HZ, and YM conceived and designed the experiments. YM performed the experiments. WC and YM wrote and revised the manuscript. All authors contributed to the article and approved the submitted version.

## Conflict of Interest

The authors declare that the research was conducted in the absence of any commercial or financial relationships that could be construed as a potential conflict of interest.

## Supplementary Material

Supplementary Figure 1 Effect of quercetin on initial attachment stage **(A)** and aggregation stage **(B)** in *S. epidermidis* biofilm formation. Biofilm formation (OD_490_) of *S. epidermidis* on initial attachment stage was detected when the cells were cultured at 37°C for 4 h with the treatment of quercetin (0 1,000 μg ml^–1^) in 96-well plates. For determining biofilm production on aggregation stage the cells were cultured at 37°C for 4 h in 96-well plates, followed by the treatment with different concentrations of quercetin (0 1,000 μg ml^–1^), and were further cultured at 37°C for 20 h. Error bars indicate the standard deviations. Statistically significant differences (determined by Student’s *t*-test) are indicated as ^∗∗∗^*P* < 0.001, ^∗∗^*P* < 0.01, and ^∗^*P* < 0.05 vs. the control group.

Supplementary Figure 2 Degradation activities of quercetin against proteins **(A)** and DNA **(B)**. Protein degradation assays were performed using milk agar plates **(A)**. Proteinase K and 5% DMSO were used as positive and negative controls. Protein digestion was observed by a clear zone surrounding proteinase K. DNA degradation of *Staphylococcus epidermidis* by quercetin was tested at 37°C for 2 h **(B)**. DNase I was used as a positive control.
