# Supplementary figures and images for: Quercetin Inhibits Biofilm Formation by Decreasing the Production of EPS and Altering the Composition of EPS in Staphylococcus epidermidis
Source: Front Microbiol. 2021 Mar 4;12:631058. doi: 10.3389/fmicb.2021.631058 (PMC7982815; doi:10.3389/fmicb.2021.631058)

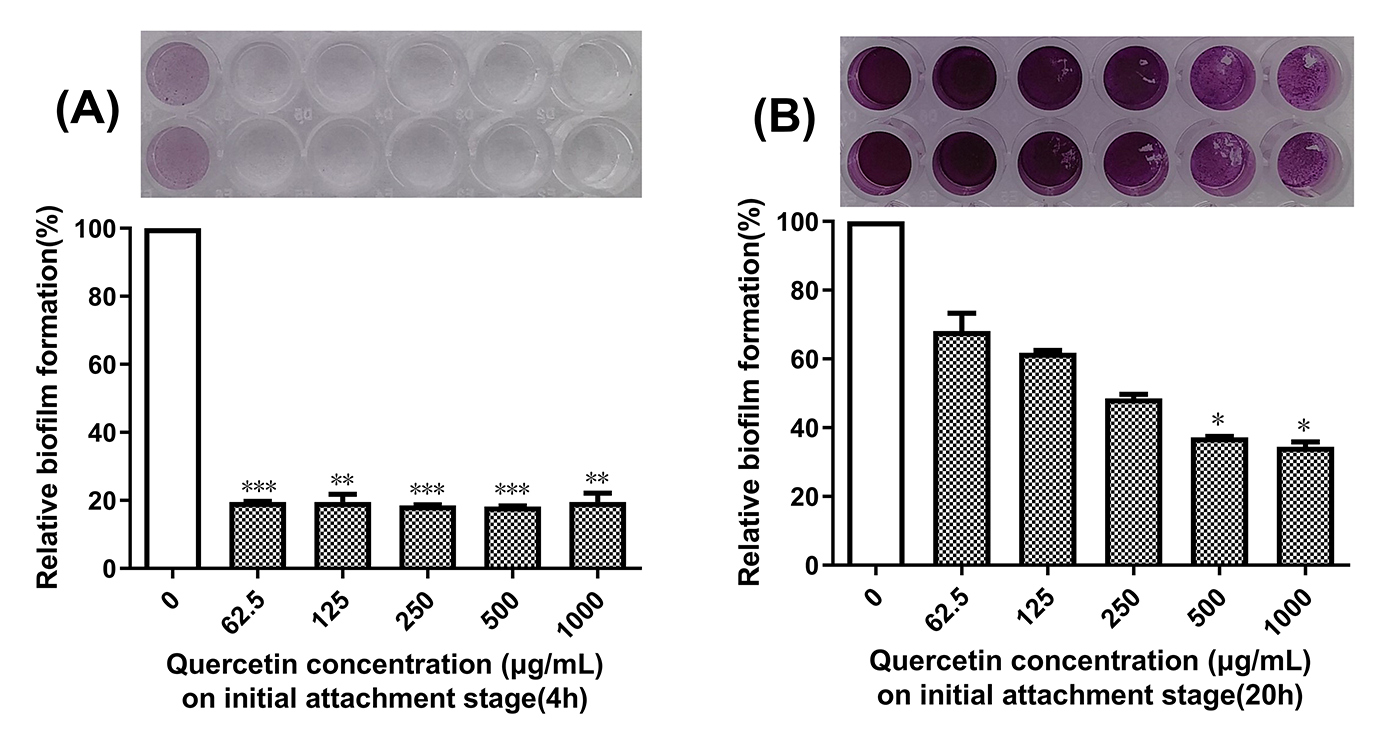

Supplement: Supplementary Figure 1 — Effect of quercetin on initial attachment stage (A) and aggregation stage (B) in S. epidermidis biofilm formation. Biofilm formation (OD490) of S. epidermidis on initial attachment stage was detected when the cells were cultured at 37°C for 4 h with the treatment of quercetin (0 1,000 μg ml–1) in 96-well plates. For determining biofilm production on aggregation stage the cells were cultured at 37°C for 4 h in 96-well plates, followed by the treatment with different concentrations of quercetin (0 1,000 μg ml–1), and were further cultured at 37°C for 20 h. Error bars indicate the standard deviations. Statistically significant differences (determined by Student’s t-test) are indicated as ∗∗∗P < 0.001, ∗∗P < 0.01, and ∗P < 0.05 vs. the control group. [file Image_1.TIF]

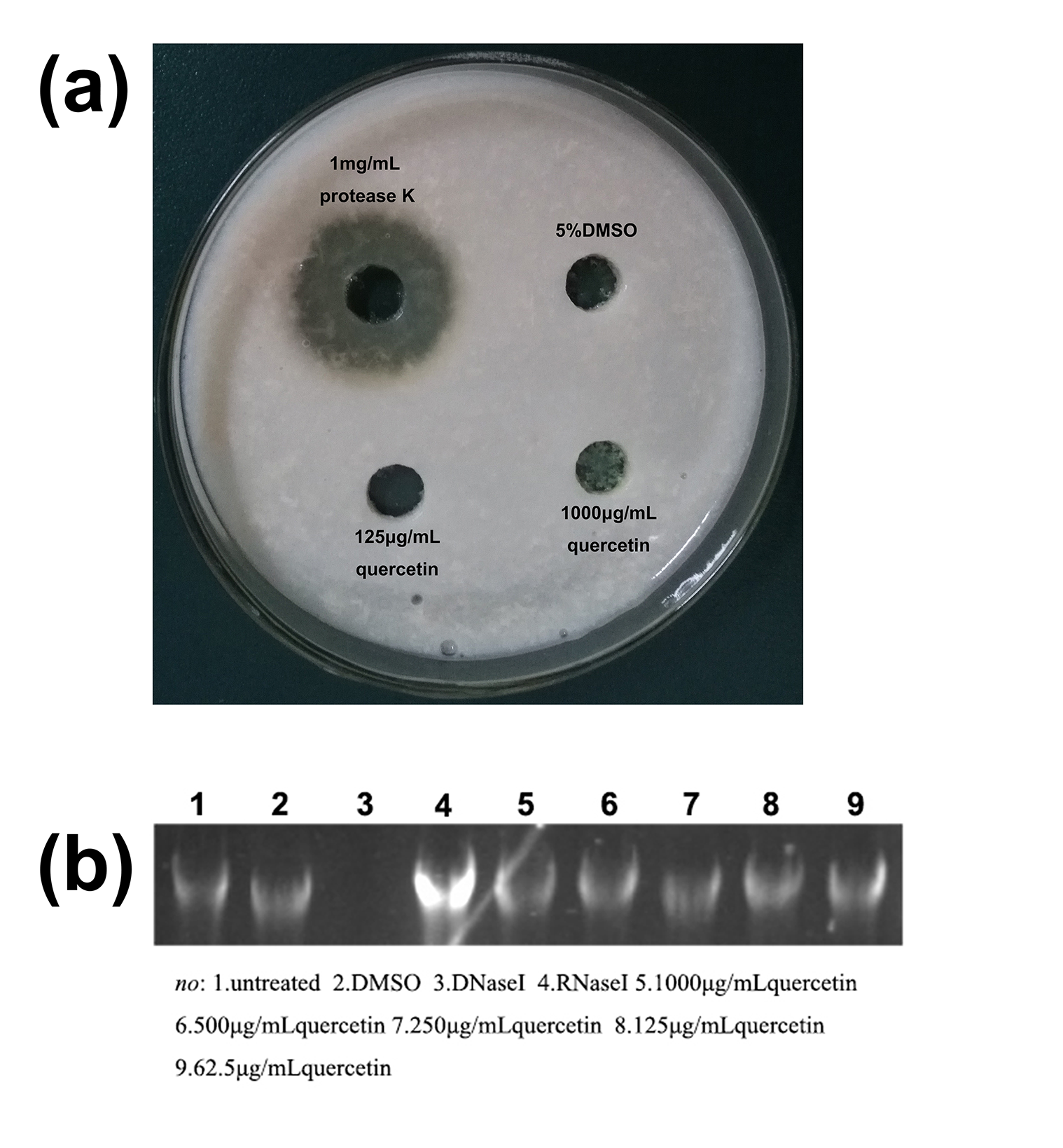

Supplement: Supplementary Figure 2 — Degradation activities of quercetin against proteins (A) and DNA (B). Protein degradation assays were performed using milk agar plates (A). Proteinase K and 5% DMSO were used as positive and negative controls. Protein digestion was observed by a clear zone surrounding proteinase K. DNA degradation of Staphylococcus epidermidis by quercetin was tested at 37°C for 2 h (B). DNase I was used as a positive control. [file Image_2.tif]
